# Assessment of jugular bulb variability based on 3D surface models: quantitative measurements and surgical implications

**DOI:** 10.1007/s00276-023-03087-x

**Published:** 2023-02-02

**Authors:** Eirik Juelke, Tobias Butzer, Abraam Yacoub, Wilhelm Wimmer, Marco Caversaccio, Lukas Anschuetz

**Affiliations:** 1 Department of Otolaryngology Head and Neck Surgery, Inselspital University Hospital and University of Bern, Bern, Switzerland; 2 Hearing Research Laboratory, ARTORG Center for Biomedical Engineering, University of Bern, Bern, Switzerland; 3 Department of Otolaryngology Head and Neck Surgery, Faculty of Medicine, Ain Shams University, Cairo, Egypt

**Keywords:** High-riding jugular bulb, Lateral skull base surgery, Three-dimensional reconstruction

## Abstract

**Purpose:**

High-riding jugular bulbs (JBs) among other anatomical variations can limit surgical access during lateral skull base surgery or middle ear surgery and must be carefully assessed preoperatively. We reconstruct 3D surface models to evaluate recent JB classification systems and assess the variability in the JB and surrounding structures.

**Methods:**

3D surface models were reconstructed from 46 temporal bones from computed tomography scans. Two independent raters visually assessed the height of the JB in the 3D models. Distances between the round window and the JB dome were measured to evaluate the spacing of this area. Additional distances between landmarks on surrounding structures were measured and statistically analyzed to describe the anatomical variability between and within subjects.

**Results:**

The visual classification revealed that 30% of the specimens had no JB, 63% a low JB, and 7% a high-riding JB. The measured mean distance from the round window to the jugular bulb ranges between 3.22 ± 0.97 mm and 10.34 ± 1.41 mm. The distance measurement (error rate 5%) was more accurate than the visual classification (error rate 15%). The variability of the JB was higher than for the surrounding structures. No systematic laterality was found for any structure.

**Conclusion:**

Qualitative analysis in 3D models can contribute to a better spatial orientation in the lateral skull base and, thereby, have important implications during planning of middle ear and lateral skull base surgery.

## Introduction

The jugular bulb (JB) is a vital vascular structure in the lateral skull base with a highly variable size and shape. In middle and inner ear surgery, a high-riding JB can limit the access to the surgical target and the maneuverability of the instruments. This can negatively affect the operative outcome and increase the risk of inadvertent injury [1, 3]. Therefore, preoperative determination of the height of the JB is of utmost importance for the planning of surgical procedures [5].

For successful surgical planning, the complete anatomical site must be taken into account. Therefore, the height of the JB is commonly classified by comparing its highest point (i.e., the dome) to the position of surrounding structures [2] or by reporting distances measured with respect to defined anatomical landmarks [2, 8, 13]. Traditionally, the measurements are taken from computed tomography (CT) scans. The assessment on CT is sometimes difficult due to the tortuous course of the JB and the surgically exposable area changes relatively rapidly with increased JB height, affecting the maneuverability of the surgical instruments. Therefore, more recent classification scales use 3D surface reconstructions of the temporal bone [7, 11]. In classification based on 3D models, the rater works with a virtual model of the anatomy and can visually compare the height of the JB to the surrounding structures. This promises a better understanding of the present anatomy and more accurate classification results. However, 3D data allowing for this new type of classification are still scarce and, thus, the variability of the JB and surrounding structures has not been sufficiently evaluated in the 3D space.

In our study, we reconstructed 3D models from 46 side-matched temporal bones of 23 subjects, applied JB classification based on these models, and examined the variability of the JB and the surrounding anatomical structures with quantitative measurements. This allowed us to describe its variability and determine surgical implications.

## Methods

### Data acquisition and 3D surface model generation

We reconstructed 46 temporal bones and the structures surrounding the JB from clinical high-resolution CT scans from subjects without temporal bone pathologies. The CT scans were obtained with a voxel size of $0.156 \times 0.156 \times 0.2$ mm$^3$ (SOMATOM Definition Edge [Siemens AG, Erlangen, Germany]). The data were processed with the threshold-based segmentation software (Amira [FEI, Bordeaux, France]) to create 3D surface models. We manually segmented the JB, the internal auditory canal (IAC), the facial nerve (FN), the semicircular canals (SCCs), the internal carotid artery (ICA), and the cochlea. The patient cohort (*n* = 23) included 10 females and 13 males (aged 20–76 years; mean age, 54.3 years). This study was approved by our institutional review board (KEK-BE 2016-00887).

### JB classification and distance measurements

We first assessed the height of the JB based on the qualitative classification presented in Manjila et al. [11]. In this classification, the height of the JB is visually compared to the height of the surrounding structures directly in the 3D model. As suggested in Hu et al. [7], we only used the major classes of JB types:

- type 1, no bulb
- type 2, below the inferior margin of the posterior semicircular canal (pSCC)
- type 3, between the inferior margin of the pSCC and the inferior margin of the IAC
- type 4, above the inferior margin of the IAC

**Fig. 1:**
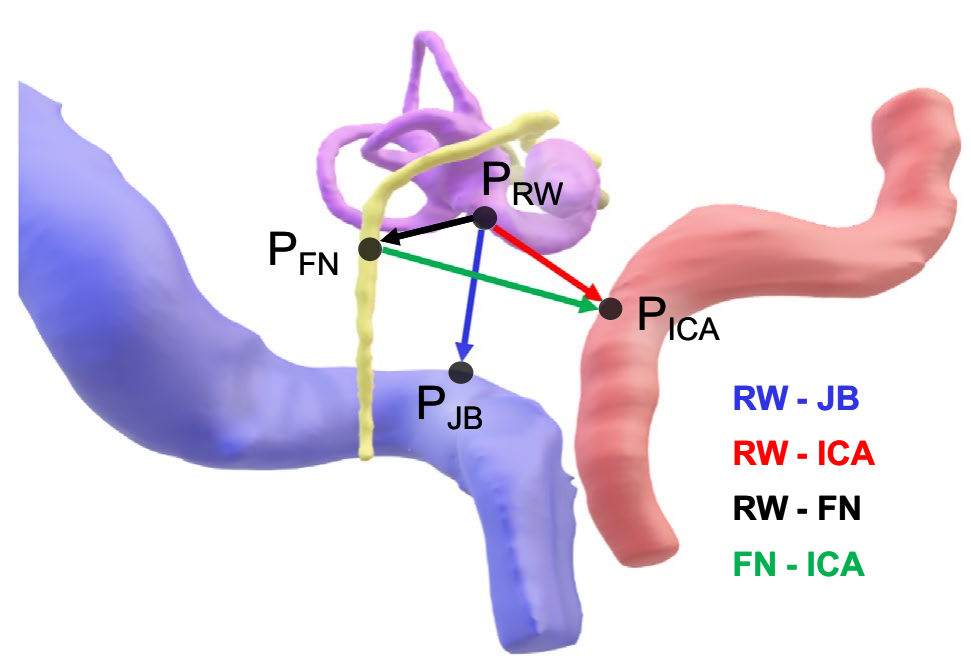
Selected anatomical landmarks and measured distances. The distance RW–JB accounts for the height of the JB. *RW* round window, *JB* jugular bulb, *ICA* internal carotid artery, *FN* facial nerve, *P*$_{\text {RW}}$ round window (main reference landmark), *P*$_{\text {JB}}$ dome of the JB, *P*$_{\text {FN}}$ FN at the level of the RW, *P*$_{\text {ICA}}$ ICA at the level of the bony annulus

For a more detailed, quantitative, description of the height of the JB, we measured its distance from surrounding structures in the 3D space. We manually identified and marked important anatomical landmarks in axial CT slices and exported their coordinates to MATLAB (MathWorks Inc., Massachusetts, USA) for automated 3D distance calculation and further analysis. We selected the round window as main reference for all distance measurements due to its importance as a constant landmark in otologic surgery [13]. The height of the JB was defined as the distance between the center of the round window and the JB dome, i.e., the most superior point of the JB [2]. To examine the variability of the most important surrounding anatomy, we measured a set of four distances (Fig. 1):

- RW-JB, distance from the center of the round window (*P*$_{\text {RW}}$) to the JB dome (*P*$_{\text {JB}}$)
- RW-FN, distance from *P*$_{\text {RW}}$ to the FN at the axial level of the round window (*P*$_{\text {FN}}$)
- RW-ICA, distance from *P*$_{\text {RW}}$ to the most posterior point of the ICA at the axial level of the bony annulus (*P*$_{\text {ICA}}$)
- FN-ICA, distance from *P*$_{\text {FN}}$ to *P*$_{\text {ICA}}$.

Two raters, (EJ, otolaryngologist, and TB, experienced scientist) performed the measurement one time each. The raters performed the measurement independently to minimize subjective measurement errors. Here, we report averaged results of the two raters.

### Anatomical differences within subjects

Besides the general anatomical variability of the structures of the middle ear, we assessed lateral differences within individual subjects. We did so by comparing distances between landmarks in the left temporal bone (DL$_{\text {left}}$) to the distances between landmarks in the right temporal bone (DL$_{\text {right}}$) for each matched pair of temporal bones (*n* = 23). We calculated the correlation between DL$_{\text {right}}$ and DL$_{\text {left}}$ and the difference $\Delta$DL between DL$_{\text {right}}$ and DL$_{\text {left}}$ to assess if and to what extent the anatomy on the right side mirrors the anatomy on the left side for individual structures.

Statistical methods used for the comparison included Pearson correlation to examine the strength of the correlation between DL$_{\text {right}}$ and DL$_{\text {left}}$ and Bayes factor analysis [9] in case of non-significant correlations. We used a Friedman test and a post hoc Nemenyi test to detect differences of $\Delta$DL among the distances RW–JB, RW–FN, RW–ICA, and ICA–FN. Statistical calculations were executed with the MATLAB statistics toolbox and the Bayes factor package [10] and R-studio [18].

## Results

### JB classification and distance measurements

**Fig. 2:**
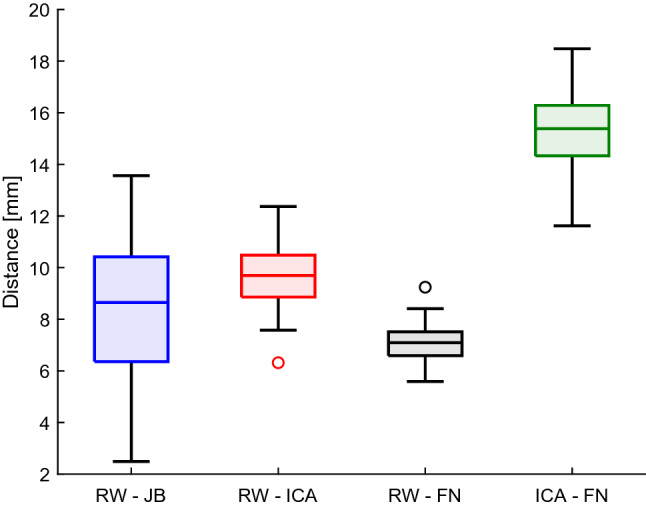
Distribution of distances between landmarks. *RW* round window, *JB* jugular bulb, *ICA* internal carotid artery, *FN* facial nerve

The examined population includes 14 sides without bulb (type 1) and 32 sides with a bulb (type 2–4). In seven cases (15%), the raters’ individual type assignment mismatched. These cases were revisited and assigned to a type by consensus. Table 1 shows the measured height of the JB (distance RW–JB) for each type separately with 30% type 1 (no identifiable bulb) and 63% type 2 (low bulb below the pSCC). The remaining distances are listed in the first column of Table 2 and the data distribution is shown in Fig. 2. RW–JB has a higher standard deviation (± 2.8mm) and larger bandwidth (between 2.5 and 13.6 mm) than the other investigated distances. This suggests a higher overall variability in the JB than in the other structures.

**Table 1:** Number of different types of JBs according to the classification of Manjila et al. [11] and the distance from the round window to the JB dome (RW–JB) for each type (mean, standard deviation, minimum, and maximum)

Classification	Nr. of sides	Distance RW–JB (mm)	
Type 1	14 (30%)	10.3 ± 1.4	(8.3; 11.9)
Type 2	29 (63%)	8.3 ± 2.5	(4.0; 13.6)
Type 3	3 (7%)	3.2 ± 1.0	(2.5; 4.3)
Type 4	None	–	–
Total	46 (100%)	–	–

### Anatomical differences within subjects

For the distance RW–ICA (*r* = 0.816, *p* <0.001), RW–FN (*r* = 0.774, *p* <0.001), and ICA–FN (0.861, *p* <0.001), we found high correlations between the right and the left side, meaning that these structures show little difference between the right and the left side. The low correlation for RW-JB (*r* = 0.345, *p* = 0.12) and Bayes factor of 1.8 suggest that the height of the JB can largely vary between the right and the left side in an individual.

**Fig. 3:**
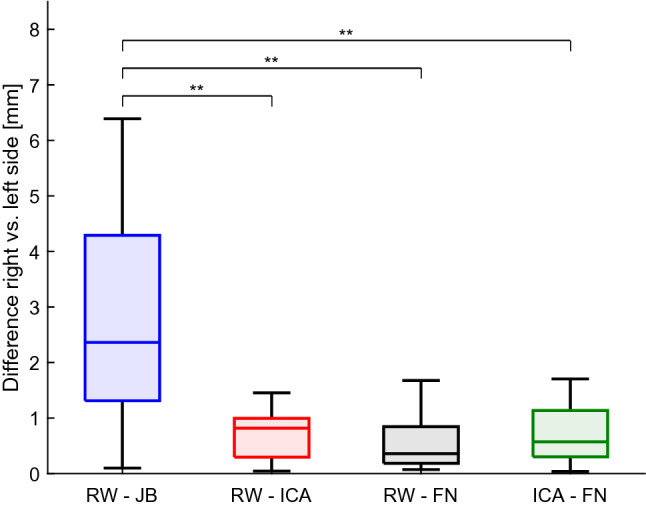
Lateral differences in distances between landmarks. *RW* round window, *JB* jugular bulb, *ICA* internal carotid artery, *FN* facial nerve. $*$Denotes significance

In terms of the extent of the difference between right and left side, statistical analysis revealed that $\Delta$DL for the height of the JB is significantly higher than $\Delta$DL$_{{\text {RW-ICA}}}$ (*p* = 0.018), $\Delta$DL$_{{\text {RW-FN}}}$ (*p* < 0.001), and $\Delta$DL$_{\text {ICA-FN}}$ (*p* = 0.013), suggesting significantly higher lateral differences for the height of the JB than for any other measured distance. Columns 2–4 of Table 2 summarize the differences of $\Delta$DL and Fig. 3 illustrates the data distribution. Interestingly, we did not find any significant dominance of the right or left side for any structure, indicating that either side can present a higher or lower JB.

**Table 2:** Distances between landmarks and lateral differences of those distances (mean, standard deviation, minimum, and maximum)

	Distance (mm)		Difference right/left (mm)		Difference right/left (%)	
RW-JB	8.2 ± 2.8	(2.5; 13.6)	2.7 ± 1.9	(0.1; 6.4)	28.2 ± 20.4	(1.2; 72.0)
RW-ICA	9.7 ± 1.3	(6.3; 12.4)	0.7 ± 0.4	(0.0; 1.4)	7.0 ± 4.5	(0.2; 18.7)
RW-FN	7.0 ± 0.8	(5.6; 9.2)	0.5 ± 0.4	(0.1; 1.7)	7.2 ± 5.7	(1.1; 23.0)
ICA-FN	15.4 ± 1.6	(11.6; 18.5)	0.7 ± 0.5	(0.0; 1.7)	4.5 ± 3.1	(0.3; 10.9)

## Discussion

In the present study, we used different assessments of the height of the JB in 3D models including a qualitative assessment classifying the JB in four types of (high riding) JBs [11] and a quantitative assessment measuring 3D distances between the round window, the JB, the ICA, and the FN. Additionally, we examined the variability of the height of the JB, the variability of the surrounding structures and lateral differences within subjects.

In the qualitative analysis, we found 30 % with no JB (type 1) and 70 % with a presence of high-riding JB types. A recent study evaluated JB types in 90 temporal bones from 77 subjects with the same classification scheme [7]. The overall presence of high-riding JBs of type 3 or higher was small, which is in line with our results. However, 65.6 % were classified as type 1 and 34.4 % as type 2 or higher. A high jugular bulb was also identified in 63% of 378 specimens [16] and in 32% of 87 specimens [20] with different classifications. We think that the discrepancy between earlier and our results may be due to the subjective application of the classification scales (e.g., of Manjila et al. [11]. The assigned type largely depends on the manually selected view angle in the 3D model. As a reference, the inter-rater error for the type classification was 15 %, while the overall inter-rater error for the measurements was 5.7 % (0.5 mm) only. Also previously, the difficult differentiation between the presence or absence of a JB has led to different interpretations of high-riding JBs [7, 15, 21]. A more precise grading for everyday clinical practice and additional orientation points for the exact spatial extension of the structures are therefore desirable. We herein present quantitative, normative data on the distance between the JB and the RW according to the classifications. In the future, this may allow a comparable classification of the JB. Moreover, these measurements allow the surgeon to readily assess the available space to operate in this delicate area of the middle ear.

In the distance measurements, we found a large variation in the JB height, ranging from distances RW–JB of as little as 2.5 mm to as much as 13.6 mm. This high variability is in line with previous studies on the height of the JB [7, 11, 14, 15, 17, 20]. For the other structures, the data distribution is much less variable. This was expected due to the high symmetry of the temporal bone [12].

The results of the assessment of the lateral differences $\Delta$DL show a high correlation (*r* > 0.7 [6]) between the right and the left side for RW–ICA, RW–FN, and FN–ICA. The difference in the distance RW–JB from the right and the left side is 28.2 % ± 20.4 %. Accordingly, the correlation between the right and the left side for the height of the JB RW–JB was low. While this result was expected due to the known variability of the JB, the low extent of the correlation (*r* = 0.345 and *p* = 0.12), indicating negligible correlation [6], was surprising. Recent work identified lateral differences including larger diameters of the jugular foramen [4] and significantly higher JBs (distance from the JB dome to the IAC) [19] on the right side. For these previous studies, it is unclear if matched sides (i.e., within subject comparison) or a general right-to-left difference was assessed. Comparing the anatomy within subjects, in our population only 52% of all subjects had higher JB on the right side. Thus, despite finding significantly higher lateral differences in the JB compared to all other structures, we did not find any side showing significantly higher JBs systematically.

Certain limitations of classification in 3D models have to be considered. First, there is a large subjective bias. We aimed to minimize such errors through two independent raters and report the inter-rater difference. Second, the number of samples, the age range, and the variation of ethnic background are limited in this study, limiting the results’ validity to cohorts similar to the cohort examined in this study.

Distance measurements in 3D models are not applicable in everyday clinical practice. However, the generation of 3D models would be feasible in selected surgical cases, where the height of jugular bulb is of utmost importance. For example in cases requiring an infracochlear access to the inferior petrous apex (cholesteatoma, cholesterol granuloma), or for cochlear implant surgery, preoperative assessment may help the surgeon to safely and efficiently plan the intervention and prevent intraoperative JB injuries. Independent of the surgical technique, 3D models displaying the exact course of the JB could be used to set up a bleeding control strategy during preoperative planning. Moreover, the generated models may be implied in stereotactic intraoperative guidance.

While classification of the JB in 3D models remains subjective, qualitative analysis in 3D models can contribute to a better spatial orientation in the lateral skull base and, thereby, have important implications during planning of middle ear and lateral skull base surgery.

## Data Availability

Please contact L. Anschuetz for data and materials: Lukas.Anschuetz@insel.ch
